# A comparative study of vacuum tumbling and immersion marination on quality, microstructure, and protein changes of Xueshan chicken

**DOI:** 10.3389/fnut.2022.1064521

**Published:** 2022-11-23

**Authors:** Qingfeng Ge, Shuyang Guo, Sheng Chen, Yuehao Wu, Zhaoyang Jia, Zhuangli Kang, Guoyuan Xiong, Hai Yu, Mangang Wu, Rui Liu

**Affiliations:** ^1^School of Food Science and Engineering, Yangzhou University, Yangzhou, Jiangsu, China; ^2^Industrial Engineering Center for Huaiyang Cuisine of Jiangsu Province, School of Tourism and Cuisine, Yangzhou University, Yangzhou, Jiangsu, China; ^3^Engineering Laboratory for Agro-Products Processing, Anhui Agricultural University, Hefei, China

**Keywords:** vacuum tumbling, immersion, protein denaturation, meat texture, water retention

## Abstract

Marination is a common technology in meat processing with advantages of enhancing tenderness, water retention, and overall quality. This study was conducted to investigate the effect of vacuum tumbling and immersion marination on meat quality, microstructure, water mobility, protein changes, and denaturation of Xueshan chicken. Results showed that vacuum tumbling significantly increased the marinating rate of chicken, tenderness, meat texture, and water retention. Meanwhile, vacuum tumbling decreased total sulfhydryl content alongside an increased protein surface hydrophobicity and free sulfhydryl content, indicating that vacuum tumbling elevated the degree of protein denaturation. Further, the peak area corresponding to the relaxation time T_22_ after vacuum tumbling was significantly higher than that of immersion marination, suggesting that the stability of the immobilized water of chicken was reduced by vacuum tumbling. Compared to immersion marination, vacuum tumbling improved myofibril fragmentation index (MFI) presenting fewer myofibrillar protein bands in sodium dodecyl sulfate polyacrylamide gel electrophoresis (SDS-PAGE) gel and more damaged muscular cells. Overall, vacuum tumbling could improve the marination absorptivity, protein degradation, and denaturation, resulting in changes in myofibril structure and meat quality of Xueshan chicken.

## Introduction

Chicken is a healthy food option that is high in protein and low in fat. It has gained increasing consumer preference due to its delicate texture and high unsaturated fatty acid content (1, 2). Inferior meat attributes are usually occurred in fast-growing broiler with pale-soft-exudative conditions, white striping, and woody breast, resulting in high water loss and low yield during processing (3, 4). Slowing-growing (SG) genotype of broiler has been perceived to have better meat quality and flavor characteristics in comparison to fast-growing broiler (5, 6). Xueshan chicken is a SG chicken crossbred by Tibetan chicken and Chahua chicken. It is a major local yellow-feathered breed for meat resource in China and shares a market value of 7.69 billion ¥ in 2020 (7). In recent years, people have become increasingly demanding of high-quality meat products. Correspondingly, the meat industry has become focused on improving quality and nutritional value of poultry meat products.

Marination has been known as an important step in meat processing. It is often used to tenderize, increase water retention, and improve overall meat quality (8, 9). Immersion marination is a traditional method of marinating and refers to the soaking of meat into marinades which are comprised of water, salts, phosphate, flavoring, and other ingredients. The immersion marination is slow and uneven in salt penetration, which affects the quality of the product. In order to make marination more efficient, a series of meat marination technologies have been developed, including muti-needle injection, ultrasound-assisted marination, massaging, tumbling, or vacuum tumbling (9–11). Tumbling is a well-known physical-mechanical treatment method, and it can rupture muscular cell membranes for marinade easily penetrated and shorten the marination time (12). A vacuum tumbler is usually utilized in the tumbling process to promote marinade penetration, leading to the more efficient entry of the marinade into the meat. Vacuum processing can not only expand the muscle fiber by different internal and external pressures, but also discharge of occluded gases of meat (13). The vacuum and mechanical movements of tumbling exhibit synergistic effects of dispersing liquids from the meat pores and extracting meat proteins to the surface. At the end of the vacuum tumbling of meat products, the atmospheric pressure would compress the residual gas in the pores and favor the infiltration of exogenous solution through the pores of meat (14). Thus, vacuum tumbling can promote the relaxation of muscle fiber structures, and make the distribution of the marinade in the meat more uniform and finally improve the quality of meat products (15, 16).

Protein denaturation generally occurs during meat processing, which has a great influence on the color and texture characteristics of meat (17). The denaturation of myofibrillar proteins may expose the internal sulfhydryl groups of protein to be oxidized to disulfide bonds, resulting in an increase in protein surface hydrophobicity and decreased protein solubility (18, 19). This affects the texture and the state of water of meat products, and these qualities play a decisive role in overall quality (20). Proteins undergo complex biochemical changes including protein degradation during the processing process which may also account for changes in the color, texture, and water retention capacity of the meat (21).

Vacuum tumbling marination has been achieved as a common technology in chicken meat processing. Previous studies were mainly focused on the various marinade ingredients and formulations (22, 23), processing variables such as carcass chilling (24), sub-sampling (25), and deboning time (26), and tumbler machine operating parameters (27) on quality attributes of chicken meat product. The swell of myofibrils and solubilization of myofibrillar proteins are thought to be responsible for the quality changes by vacuum tumbling (8) while no evidence has been found in chicken meat processing. To our knowledge, little information is available on the effect of vacuum tumbling on changes in protein degradation and denaturation, the microstructure of muscle cells and mobility of water, which were accounting for quality attributes of chicken. Thus, the aim of this study was to investigate the effect of vacuum tumbling on the quality characteristics, water holding capacity, and protein changes of Xueshan chicken compared with immersion marination, to explore the relationship between protein denaturation and quality characteristics.

## Materials and methods

### Sample preparation

The chilled Xueshan chicken (3-month-old, weight of 1.20 ± 0.05 kg) was purchased from Lihua livestock Co. Ltd. (Changzhou, Jiangsu, China) and was thawed at 4°C for 24 h. The marinade was prepared according to the weight of the chicken, and the volume ratio of liquid to meat was 1:1. The marinade ingredients included pepper juice (0.05%, w/w), spiced juice (0.08%, w/w), ethyl maltol (0.04%, w/w), sodium glutamate (0.15%, w/w), sand ginger powder (0.2%, w/w), sodium chloride (6.41%, w/w), and sodium tripolyphosphate (0.42%, w/w). The pepper juice and spiced juice were purchased from Tieling Hongyuan Food Co. Ltd. (Liaoning, China). There were 30 whole chicken carcasses which were randomly allocated into tumbling marination treatment and immersion marination treatment (15 chickens per treatment) and performed three batches (five chickens per batch) using a vacuum tumbler (ESK125, Vakona Gmbh Co., Ltd., Lienen, Germany). The parameters of tumbling marination were set from our trial tests considering the marinating absorptivity and sensory evaluation of Xueshan chicken. The marination time was set for 10 h at a speed of seven revolutions per min. The vacuum was set for −0.06 MPa, in a cycle of 20 min on and 10 min off at 4°C. The chicken by immersion marinating was immersed in the marinade for 15 h at 4°C, which was performed by the manufacturer’s recommendation from Lihua livestock Co. Ltd. After completion of marinating treatments, chicken breasts were obtained for quality determination and protein measurements.

### Determination of marinating absorptivity and pH

The marination absorptivity was measured according to the method of Peiretti et al. (28). The weight of chicken breast before marination was recorded as m_1_ and the weight of marinated chicken breast was recorded as m_2_. The marination absorptivity was calculated as the percentage of difference of m_2_ and m_1_ over m_1_. The pH of the sample was measured using a portable pH meter (FE20K; Mettler Toledo, Zurich, Switzerland). The probe was inserted into the chicken breast, and each sample was measured at four different points and averaged.

### Meat quality measurements

The color attributes including L*, a*, and b* values on the chicken breast surface were measured by a colorimeter (CR-400; Konica Minolta, Tokyo, Japan). The press loss and cooking loss were measured according to the method of Kauffman et al. (29) and Xia et al. (30), respectively, and expressed as the percentage of weight difference over the initial weight. The shear force was measured by the method described by Chen et al. (31). After the determination of cooking loss, the sample was cut along the direction of the muscle fiber, and the meat slices were cut with a C-LM3B digital display tenderness meter (Northeast Agricultural University, Haerbin, China). The texture profile analysis was conducted using a texture analyzer (TA-XT Plus, Stable Micro System, Surrey, UK) according to Malila et al. (32) with slight modifications. The cooked chicken breast was cooled and cut into 1 × 1 × 1 cm^3^ pieces along the direction of the muscle fibers. The texture profiles including hardness, springiness, cohesiveness, chewiness, and resilience were analyzed by Texture Expert Exceed 2.64a.

### Nuclear magnetic resonance transverse relaxation (T_2_) analysis

The water distribution and proportion in the chicken breast meat samples were determined using a micro nuclear magnetic resonance (NMR) instrument (Meso MR23, Niumag Electric Corporation, Shanghai, China) by the method of Kang et al. (33) with some modifications. The meat sample was cut into a 1 cm diameter with a length of 2 cm along the direction of the muscle fiber and placed in a glass magnetic resonance tube with a diameter of 1 cm. The spin-selective relaxation time (T_2_) was measured using the Carr–Purcell–Meiboom–Gill (CPMG) pulse sequence, and the instrument temperature was stable at 32°C. The measurement parameters were set as follows: TR = 4,500 ms, SW = 100 KHz, NECH = 4,000, NS = 32, τ = 200 μs, and the obtained attenuation curve was inverted by Multi-Exp Inv analysis software. Statistical analysis was performed on the proportion of moisture content, and each test was repeated three times.

### Analysis of microstructure

The microstructures of chicken breast were examined by the scanning electron microscopy (GeminiSEM 300, Carl Zeiss, Oberkochen, Germany). The marinated sample was cut into 2 cm × 0.5 cm × 0.5 cm pieces vertical to the muscle fiber direction and fixed in 2.5% glutaraldehyde dissolved in 0.1 M phosphate buffer (pH 7.2) overnight at 4°C. The specimens were washed twice with 0.1 M phosphate buffer (pH 7.2) and each for 15 min. The samples were dehydrated with a gradient of 30%, 50%, 70%, 80%, 90%, 95%, 100% (two times), 100% (with anhydrous sodium sulfate) ethanol, each for 15 min. The specimens were dried with a critical point dryer (CPD-300, Leica, Heerbrugg, Switzerland) and coated with gold (SCD 500, BAL-TEC, Vienna, Austria).

### Determination of myofibrillar protein solubility

Meat sample (2 g) was homogenized three times with 40 ml of 0.1 M ice-cold potassium phosphate buffer (pH 7.2, 1.1 M potassium iodide) to extract total protein. The samples were then placed on a shaker and extracted at 4°C for 12 h. The samples were then centrifuged (1,500 *g*, 20 min) at 4°C and the BCA protein assay kit (Thermo Scientific, Rockford, USA) was used to determine the concentration of supernatant protein. Sarcoplasmic protein solubility was measured by 0.025 M ice-cold potassium phosphate buffer (pH 7.2), and performed the same extraction and BCA measurement protocol. The myofibrillar protein solubility was expressed as the difference between total protein solubility and sarcoplasmic protein solubility.

### Preparation of myofibrillar protein

The extraction of myofibrillar protein was carried out according to Park and Xiong (34) with slight modifications. The chopped meat (10 g) was mixed with 4 vol of separation buffer (10 mM phosphate buffer, 2 mM MgCl_2_, 0.1 M NaCl, and 1 mM EGTA, pH 7.0), homogenized for 30 s and centrifuged (4°C, 2,000 × *g*, 15 min). The supernatant was discarded and the pellet was washed two more times with 4 vol of the separation buffer. The pellet was then washed three times with 4 vol of 0.1 M NaCl. The supernatant was discarded and the myofibrillar protein was obtained to detect protein concentration by Biuret protein assay. Then, the myofibrillar protein was stored at 4°C for use within 24 h.

### Determination of protein surface hydrophobicity

The myofibrillar protein was dissolved in 20 mM phosphate buffer (containing 0.01 M K_2_HPO_4_, pH 7.0) and diluted to a concentration of 1.5 mg/ml. The 20 μl of 8 mM 8-Anilino-1-naphthalene sulfonic acid (ANS) was added to 2 ml of the diluted solution and incubated at room temperature for 10 min. The fluorescence intensity of the protein was determined at an excitation wavelength of 380 nm and an emission wavelength of 490 nm. ANS was not added to the blank. The different protein concentrations were taken as the abscissa and the corresponding fluorescence intensity was plotted as the ordinate. The surface hydrophobicity of protein was represented by the slope of the regression fit curve, and the initial slope was called S_0_ANS.

### Determination of protein sulfhydryl content

Protein sulfhydryl content was determined by the method described by Fu et al. (35), with minor modifications. The 1 ml of 2 mg/ml myofibrillar protein solution was mixed with 9 ml of 50 mM PBS (0.01 M EDTA, 0.6 M KCl, 8 M urea, 0.02 M phosphate buffer, pH 7.0) and 0.4 ml of 10 mM 5,5′-dithiobis (2-nitrobenzoic acid) DTNB. The mixture was incubated at 40°C for 25 min. The absorbance of the sample was measured at 412 nm with a molar extinction coefficient of 13,600 M^–1^ cm^–1^. The free sulfhydryl content was determined by incubation at 4°C for 1 h in a urea-free reaction mixture. Protein sulfhydryl content was expressed in nmol/mg protein.

### Determination of myofibril fragmentation index

The myofibril fragmentation index (MFI) was determined according to the method of Culler et al. (36). The myofibrillar protein solution was adjusted to 0.5 mg/ml and its absorbance at 540 nm was measured using a microplate reader (Spectra Max M3; Molecular Devices, Sunnyvale, USA). The MFI was obtained by an absorbance value multiplying by 200.

### Sodium dodecyl sulfate polyacrylamide gel electrophoresis

The concentration of myofibrillar protein was adjusted to 4 mg/ml and mixed with the loading buffer [10 mM *Tris–HCl*, 2.5% (m/v) SDS, 1% (v/v) β-mercaptoethanol, 10% glycerol (v/v), and 0.01% (m/v) bromophenol blue, pH 6.8] in an equal volume, followed by heating in a water bath at 95°C for 5 min. The separating gel was 10%. The initial electrophoresis voltage was 90 V, and after 30 min, the voltage was increased to 120 V for 60 min. After electrophoresis, the gel was stained with a staining buffer containing 45% methanol, 10% glacial acetic acid, and 0.1% (m/v) coomassie brilliant blue, and then de-stained with distilled water before scanning on a gel imager (GT-800F, Epson, Nagano, Japan).

### Statistical analysis

All analytical tests were performed in triplicate. The data were statistically analyzed using SPSS 16.0 software and the results were expressed as mean ± standard error (SE). The *t*-test was used to compare two treatments, and the significant difference level was *P* < 0.05.

## Results and discussion

### Water holding capacity and pH

The water retention of chicken after marination is shown in Table 1. The marination absorptivity of the chicken breast marinated by vacuum tumbling was higher than that of immersion marinating (*P* < 0.01). It was previously reported that the addition of sodium tripolyphosphate promoted the dissociation of actomyosin to myosin in meat, which had a strong water retention capacity, thereby increasing the water retention of chicken (8, 37). The mechanical action of tumbling marination promoted relaxation of the muscle tissue, and the fascia tissue was partially destroyed, allowing the marinade to penetrate into the meat more quickly (9, 13). The widening of muscle fiber gaps allowed the chicken to maintain more water. A large amount of the marination liquid was absorbed by the chicken, which caused the cooking loss of the tumbling marinated chicken to be significantly higher than that of immersion marination (*P* < 0.05). However, with respect to press loss, there was no significant difference between immersion marination and vacuum tumbling (*P* > 0.05), indicating a comparable effect of water holding capacity in chicken meat after two marination treatments. The pH value has a great influence on the quality of the meat. Compared to immersion marination, tumbling marination significantly increased the pH value of chicken breast (*P* < 0.05) possibly due to the higher absorptivity of marinade, which contained alkaline tripolyphosphate (38, 39). During the marination process, sodium chloride and sodium tripolyphosphate in the marinade diffused into the muscle tissue due to osmotic pressure, resulting in an increase in the pH of the chicken breast.

**TABLE 1:** Effect of vacuum tumbling and immersion marinating on chicken meat quality attributes.

Sample	Immersion marinating	Tumbling marinating	*P-value*
Absorptivity/%	4.91 ± 0.21	28.92 ± 0.81	<0.001
Cooking loss/%	9.44 ± 0.47	11.32 ± 0.53	<0.01
Press loss/%	21.91 ± 0.58	22.41 ± 0.87	0.262
pH	5.620.03	5.81 ± 0.04	<0.001
*L* *	39.61 ± 1.16	43.52 ± 1.08	<0.001
*a* *	7.35 ± 0.79	6.07 ± 0.55	<0.001
*b* *	12.22 ± 1.02	14.93 ± 2.71	0.018
Hardness/g	2788 ± 115	1925 ± 89	<0.001
Springiness/mm	0.69 ± 0.02	0.71 ± 0.02	0.014
Cohesiveness/Ratio	0.58 ± 0.04	0.46 ± 0.03	<0.001
Chewiness/mJ	1212 ± 98	783 ± 76	<0.001
Shear force/N	41.95 ± 1.04	27.12 ± 0.98	<0.001

The L*, a* and b* indicated lightness, redness and yellowness, respectively, which were color attributes.

### Color

Color is an important sensory quality of meat. The effect of marination methods on the color of chicken breast meat is shown in Table 1. The L* of the chicken meat marinated by vacuum tumbling was higher (*P* < 0.01) than after immersion marination. The L* is related to the water retention of the meat. An increase in the L* by vacuum tumbling was due to the fact that muscle cells absorb a large amount of water during the marination process, which changes the reflection characteristics of the meat, resulting in a higher L* of marinated chicken (40).

Vacuum tumbling group showed a significantly lower a* value and higher b* value than that of the immersion margination group (Table 1, *P* < 0.01). During the vacuum tumbling process, the mechanical effect of the tumbling treatment may disrupt the muscle fibers, resulting in the change in myoglobin content in muscle cell (41). Moreover, the myoglobin in the chicken might be oxidized to oxygenated myoglobin, and the long-term vacuum tumbling marination caused the oxygenated myoglobin further oxidized to form brown metmyoglobin, leading to a decrease in the observed a* value. In addition, as the tumbling time increased, the residual blood in the meat gradually flowed out, which also caused a* of chicken breast meat to decrease. The b* of tumbling marinated chicken breast was shown to be significantly higher than that of the immersion treatment in the current study, which may be caused by protein denaturation during the rolling process (40).

### Texture and tenderness of chicken breast

Texture and tenderness are important indicators for evaluating meat quality and have a great influence on the taste of meat. The tenderness of meat is usually expressed by shear force. As presented in Table 1, hardness and chewiness of the chicken prepared by vacuum tumbling were lower than after immersion marination (*P* < 0.01). Consistently, the shear force of tumble-marinated chicken breast was lower than immersion marination (*P* < 0.01). The mechanical effect of vacuum tumbling treatment may disrupt the muscle fibers and cause the muscle tissue to be loosen, thereby promoting the diffusion of the marinade into the muscle tissue. In addition, under vacuum conditions, the muscle fibers swelled due to the difference between internal and external pressure, which increased the gap between the muscle fibers and promoted the structural relaxation of the muscle fibers (13). It was shown that the cohesiveness of meat was related to the integrity of muscle fiber structure and greater cohesiveness indicated less damaged muscle fiber. The cohesiveness of chicken marinated by vacuum tumbling was significantly lower than after immersion marination (*P* < 0.01), which indicated more damage to muscle fibers by vacuum tumbling. Moreover, mechanical effects and vacuum may cause the release of endogenous proteases such as cathepsins and calpains in meat, thereby further increasing the tenderness of chicken (42, 43). As a result, chicken marinated by vacuum tumbling had significantly higher tenderness compared to immersion marination.

### NNR transverse relaxation (T_2_)

**FIGURE 1:**
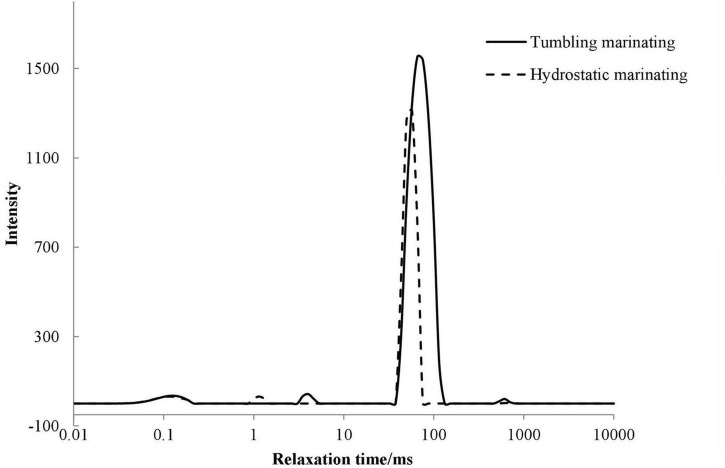
Effects of vacuum tumbling and immersion marinations on the T_2_ relaxation time (ms) of prepared chicken breast.

Nuclear magnetic resonance transverse relaxation can reflect the mobility of water in food matrix (44). As shown in Figure 1, four water groups were observed and included T_20_ (0–0.1 ms), T_21_ (1–10 ms), T_22_ (10–100 ms), and T_23_ (100–1,000 ms). T_20_ and T_21_ represented water that was tightly bound to macromolecules, where T_20_ was strongly bound water and T_21_ was weakly bound water. T_22_ indicated the immobilized water inside the organized protein structure, and T_23_ was referred to as the free water outside the myofibril.

As shown in Table 2, no significant difference was found in the peak time of T_20_ between immersion and vacuum tumbling marination (*P* > 0.05). However, vacuum tumbling marination significantly decreased the combined water peak area ratio of P_20_ and P_21_ (*P* < 0.05), which may be related to the change in protein spatial structure. Compared with immersion marination, the vacuum tumbling marination significantly increased the relaxation time of T_21_, T_22_, and T_23_, and the peak area of T_22_ (*P* < 0.05). It was suggested that vacuum tumbling marination significantly increased the immobilized water content of the chicken. This may be due to the mechanical effect of vacuum tumbling, which led to the increase in muscle fiber gap and more water absorption inside muscular cells, thereby raising the content of the immobilized water. The vacuum tumbling group had a higher T_22_ value than the immersion group. The vacuum tumbling marination treatment might destroy the intact structure of the muscle fiber and reduce the stability of the immobilized water. In addition, vacuum tumbling marination was able to absorb much-marinating liquid and then increased moisture content and water mobility (33).

**TABLE 2:** Effect of vacuum tumbling and immersion marination on water mobility and distribution of chicken breast.

Samples	Distribution of T2 relaxometry time/ms				Peak area ratio of T2 relaxometry time/%			
	T_20_	T_21_	T_22_	T_23_	P_20_	P_21_	P_22_	P_23_
Immersion marinating	0.14 ± 0.06	1.010 ± 0.12	57.22 ± 2.15	786.48 ± 42.15	5.16 ± 0.13	1.69 ± 0.22	91.40 ± 1.02	0.18 ± 0.10
Tumbling marinating	0.14 ± 0.08	4.037 ± 0.31	65.79 ± 2.49	464.16 ± 33.01	2.20 ± 1.20	1.17 ± 0.16	96.19 ± 1.34	0.44 ± 0.04
*P*-value	0.801	<0.001	<0.001	<0.001	<0.001	0.017	0.004	<0.001

### Protein properties

The MFI is inversely related to the integrity of the internal structure of myofibrils and affects the tenderness of the meat (45). As seen in Table 3, the MFI value of the chicken marinated by vacuum tumbling significantly increased (*P* < 0.05) compared to immersion marination. During the vacuum tumbling process, the mechanical effects caused the breakage of muscle fiber and improved tenderness, which was consistent with the shear force measurement (Table 1).

**TABLE 3:** Effect of vacuum tumbling and immersion marination on protein properties.

Parameters	Immersion marinating	Tumbling marinating	*P-value*
Myofibril fragmentation index	22.71 ± 0.11	25.52 ± 0.19	<0.001
Protein surface hydrophobicity	1035.67 ± 3.75	1275.63 ± 28.97	<0.001
Total sulfhydryl/nmol mg^–1^ protein	140.43 ± 1.73	119.37 ± 3.04	<0.001
Free sulfhydryl/nmol mg^–1^ protein	97.54 ± 2.49	109.87 ± 2.23	<0.001
Total protein solubility/mg g^–1^	225.46 ± 5.86	195.38 ± 4.35	<0.001
Sarcoplasmic protein solubility/mg g^–1^	102.27 ± 2.91	98.54 ± 3.65	0.059
Myofibrillar protein solubility/mg g^–1^	122.59 ± 2.63	96.84 ± 3.85	<0.001

The surface hydrophobicity of the protein reflects the relative content of hydrophobic residues on the surface of the protein molecule and is related to protein functional properties (46, 47). The greater surface hydrophobicity of myofibrillar proteins indicated more exposed the hydrophobic residues inside the protein molecules. As shown in Table 3, the protein surface hydrophobicity of the chicken marinated by vacuum tumbling was significantly higher than that by immersion marination (*P* < 0.05). Protein denaturation can lead to the exposure of hydrophobic groups inside the protein (19, 48). It was implied that vacuum tumbling caused denaturation of myofibrillar proteins, leading to the exposure of hydrophobic residues inside the protein, which increased the surface hydrophobicity of the protein.

The total sulfhydryl content of the vacuum tumbling marination group was lower than immersion marination (*P* < 0.05). The continuously physico-mechanical action (e.g., friction and beating) of tumbling marination as well as the catalysis of metal ions in the chamber wall of a tumbling machine were considered as important factors for the oxidation of meat proteins. Free sulfhydryl groups are closely related to the distribution and content of sulfhydryl groups on the surface of proteins. As shown in Table 3, the free sulfhydryl content of the vacuum tumbling marination group was higher than immersion marnation group (*P* < 0.05), indicating that vacuum tumbling marination increased the degree of protein denaturation. It was shown that the free sulfhydryl content of protein was positively correlated with surface hydrophobicity (49). Vacuum tumbling marination was speculated to denature meat proteins, causing structural changes, and exposing hydrophobic groups which were embedded inside the protein. Moreover, the change of protein spatial structures led to the exposure of sulfhydryl groups, increasing the content of free sulfhydryl groups, and this change of free sulfhydryl content was consistent with the surface hydrophobicity.

The solubility of proteins is closely related to their functional properties, reflecting some extent the degree of denaturation of proteins. The effect of two marination methods on protein solubility is shown in Table 3. The total protein solubility of the vacuum tumbling group was lower (*P* < 0.05) than that of immersion group. Vacuum tumbling had a greater effect on the solubility of total protein, indicating more denaturation of chicken protein compared to immersion marination. During the vacuum tumbling marination process, salt and sodium tripolyphosphate can promote the dissolution of salt-soluble proteins, resulting in a decrease in the solubility of myofibrillar proteins. Further, the vacuum conditions and the mechanical effects of tumbling accelerated the dissolution of salt-soluble proteins, which may also result in a significantly lower myofibrillar solubility in the vacuum tumbling group.

### Microstructure

**FIGURE 2:**
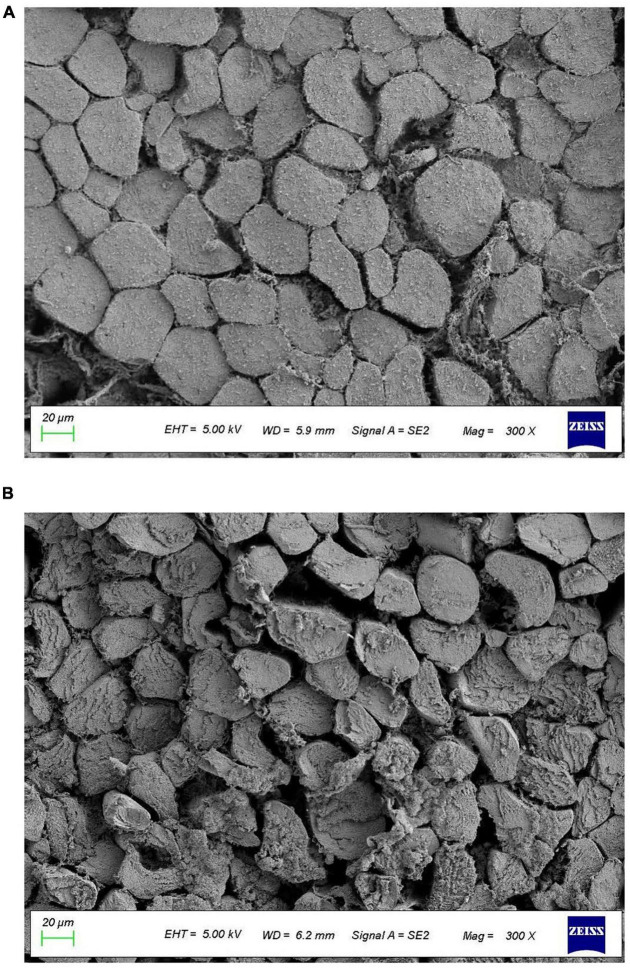
Effects of vacuum tumbling **(A)** and immersion marinations **(B)** on the microstructure of chicken.

As it was shown in Figure 2, the muscular cells in the immersion group were closely arranged, and the gap between muscle fibers was small, indicating that the degree of damage to the muscle fibers by immersion was limited. However, the muscular cells in vacuum tumbling marination group was seen to be severely damaged and the muscle bundle membrane was broken to disrupt the integrity of muscle fibers. Moreover, the gap between the muscle fibers was visibly larger by vacuum tumbling marination, compared to immersion marination. This indicates that vacuum tumbling destroyed the structure of the muscle fibers, increased the gap of the muscle fibers, and loosened the structure of the muscle fibers, thereby attributing to the tenderness promotion and also the protein denaturation of chicken meat by vacuum tumbling (50).

### SDS-PAGE

**FIGURE 3:**
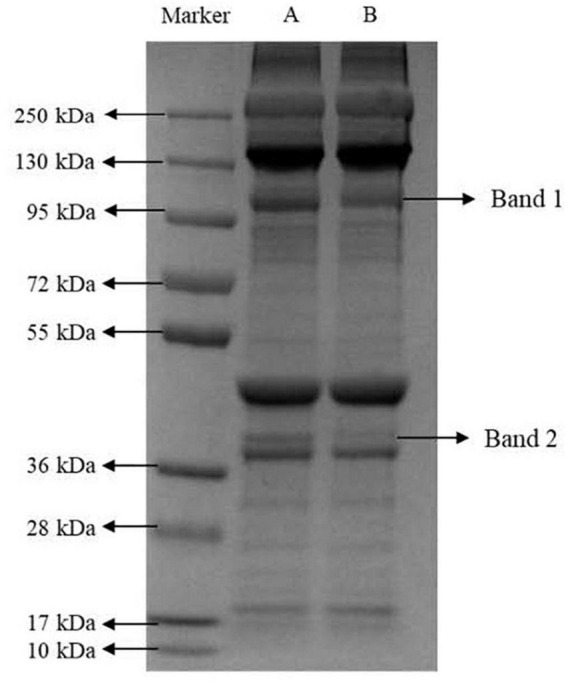
Representative image of SDS-PAGE gels of myofibrillar proteins in chicken breast by marinating treatment. The chicken breasts treated with immersion and vacuum tumbling marinating were denoted as A and B, respectively.

The effect of marination on the myofibrillar protein bands is shown in Figure 3. Compared with immersion marination, the intensity of the protein bands at the Band 1 (95–130 kDa, e.g., C-protein or α-actinin) and Band 2 (36–55 kDa, e.g., tropomyosin or troponin-T) in the vacuum tumbling marination group significantly reduced, among which Band 2 was less detectable. The difference in the relative intensity of the myofibrillar protein bands maybe due to denaturation of the protein during vacuum tumbling marination, resulting in protein fragmentation. Combined with the results of total sulfhydryl content and surface hydrophobicity in the current study, it appears that vacuum tumbling promotes protein degradation and increases the degree of protein denaturation, finally affecting the quality attributes of marinated chicken meat.

## Conclusion

The study explored the effects of vacuum tumbling and immersion marination on the overall quality, microstructure, water mobility, protein changes, and denaturation of Xueshan chicken. Overall, the vacuum tumbling had more damaged muscle fiber and higher marination absorption rate than that of immersion marination, resulting in desired meat tenderness, water retention, and texture characteristics of chicken. Accordingly, vacuum tumbling significantly decreased the total sulfhydryl content and protein solubility, and increased the surface hydrophobicity and free sulfhydryl content compared to immersion marination. These results demonstrated that vacuum tumbling significantly increased the degree of protein denaturation and potentially improved the quality of marinated chicken.

## Data availability statement

The original contributions presented in this study are included in the article/supplementary material, further inquiries can be directed to the corresponding author.

## Author contributions

QG and RL: conceptualization, resources, and writing – review and editing. SC: methodology. ZJ: software. SG and YW: validation. GX: formal analysis. SC and SG: investigation. ZK: data curation. QG: writing – original draft preparation, project administration, and funding acquisition. MW: visualization. HY: supervision. All authors have read and agreed to the published version of the manuscript.

## References

[1] Sáyago-ayerdi S Brenes A Viveros A Goñi I. Antioxidative effect of dietary grape pomace concentrate on lipid oxidation of chilled and long-term frozen stored chicken patties. *Meat Sci.* (2009) 83:528–33. 10.1016/j.meatsci.2009.06.038 20416664

[2] Erdaw MM Beyene WT. Trends, prospects and the socio-economic contribution of poultry production in sub-Saharan Africa: a review. *Worlds Poult Sci J.* (2022) 78:835–52.

[3] Huang X Ahn D. The incidence of muscle abnormalities in broiler breast meat -a review. *Korean J Food Sci Anim Resour.* (2018) 38:835–50. 30479493 10.5851/kosfa.2018.e2 PMC6238037

[4] Wang YH Lin J Wang J Wu SG Qiu K Zhang HJ The role of incubation conditions on the regulation of muscle development and meat quality in poultry. *Front Physiol.* (2022) 13:883134. 10.3389/fphys.2022.883134 35784883 PMC9240787

[5] Weng KQ Huo WR Li Y Zhang Y Zhang Y Chen GH Fiber characteristics and meat quality of different muscular tissues from slow-and fast-growing broilers. *Poult Sci.* (2022) 101:101537. 10.1016/j.psj.2021.101537 34788716 PMC8591497

[6] Weng KQ Li Y Huo WR Zhang Y Cao ZF Zhang Y Comparative phosphoproteomic provides insights into meat quality differences between slow- and fast-growing broilers. *Food Chem.* (2022) 373:131408. 10.1016/j.foodchem.2021.131408 34710681

[7] Wang LD Kong LL Hu XD Bai H Wang ZX Jiang Y Effect of stocking density on performance, meat quality and cecal bacterial communities of yellow feather broilers. *Anim Biotechnol.* (2021) 22:1–11. 10.1080/10495398.2021.1898413 33752552

[8] Alvarado C McKee S. Marination to improve functional properties and safety of poultry meat. *J Appl Poult Res.* (2007) 16:113–20.

[9] Zhou YJ Hu MQ Wang L. Effects of different curing methods on edible quality and myofibrillar protein characteristics of pork. *Food Chem.* (2022) 387:132872. 10.1016/j.foodchem.2022.132872 35390604

[10] Xiong GY Fu XY Pan DM Qi J Xu XL Jiang XJ. Influence of ultrasound-assisted sodium bicarbonate marination on the curing efficiency of chicken breast meat. *Ultrason Sonochem.* (2020) 60:104808. 10.1016/j.ultsonch.2019.104808 31568999

[11] Yusop SM O’Sullivan MG Kerry JP. Marinating and enhancement of the nutritional content of processed meat products. 1st ed. In: Kerry JP Kerry JF editors. *Processed Meats: improving Safety, Nutrition and Quality.* (Vol. 66) (Philadelphia, PA: Woodhead Publishing Limited) (2011). p. 421–49. 10.1016/j.dib.2019.104801

[12] Conde E Gaillard J Núñez C Piai M Ramallo AV. Towards the string dual of tumbling and cascading gauge theories. *Phys Lett B.* (2012) 709:385–9.

[13] Zhu C Yin F Tian W Zhu Y Zhao L Zhao G. Application of a pressure-transform tumbling assisted curing technique for improving the tenderness of restructured pork chops. *LWT Food Sci Technol.* (2019) 111:125–32.

[14] Bosse R Thiermann N Gibis M Schmidt H Weiss J. Effect of mechanical curing treatments on particle distribution to simulate non-motile bacteria migration in cured raw ham. *J Food Eng.* (2017) 194:58–66. 10.1016/j.jfoodeng.2016.09.005

[15] Szerman N Gonzalez C Sancho AM Grigioni G Carduza F Vaudagna SR. Effect of whey protein concentrate and sodium chloride addition plus tumbling procedures on technological parameters, physical properties and visual appearance of sous vide cooked beef. *Meat Sci.* (2007) 76:463–73. 10.1016/j.meatsci.2007.01.001 22060988

[16] Bosse R Müller A Gibis M Weiss A Schmidt H Weiss J. Recent advances in cured raw ham manufacture. *Crit Rev Food Sci Nutr.* (2018) 58:610–30. 10.1080/10408398.2016.1208634 27469301

[17] Zhan X Sun D Zhu Z Wang Q. Improving the quality and safety of frozen muscle foods by emerging freezing technologies: a review. *Crit Rev Food Sci Nutr.* (2018) 58:2925–38. 10.1080/10408398.2017.1345854 28723226

[18] Benjakul S Visessanguan W Thongkaew C Tanaka M. Comparative study on physicochemical changes of muscle proteins from some tropical fish during frozen storage. *Food Res Int.* (2003) 36:787–95.

[19] Li YP Zhang XH Lu F Kang ZL. Effect of sodium bicarbonate and sodium chloride on aggregation and conformation of pork myofibrillar protein. *Food Chem.* (2021) 350:129233. 10.1016/j.foodchem.2021.129233 33592363

[20] Ishiwatari N Fukuoka M Sakai N. Effect of protein denaturation degree on texture and water state of cooked meat. *J Food Eng.* (2013) 117:361–9.

[21] Yu Q Wu W Tian X Jia F Xu L Dai R Comparative proteomics to reveal muscle-specific beef color stability of holstein cattle during post-mortem storage. *Food Chem.* (2017) 229:769–78. 10.1016/j.foodchem.2017.03.004 28372243

[22] Petracci M Laghi L Rimini S Rocculi P Capozzi F Cavani C. Chicken breast meat marinated with increasing levels of sodium bicarbonate. *J Poult Sci.* (2014) 51:206–12. 10.3382/ps/peu063 25589078

[23] Popat DS Manohar GR Ramamurthy N Babu RN Mir NA Jaywant RJ. Effect of vacuum tumbling on quality and economy of tandoori chicken prepared from commercial broiler chicken meat. *Indian J Poult Sci.* (2017) 52:322–6.

[24] Perumalla AVS Saha A Lee Y Meullenet JF Owens CM. Marination properties and sensory evaluation of breast fillets from air-chilled and immersion-chilled broiler carcasses. *Poult Sci.* (2011) 90:671–9. 10.3382/ps.2010-00845 21325241

[25] Bowker B Zhuang H. Freezing-thawing and sub-sampling influence the marination performance of chicken breast meat. *Poult Sci.* (2017) 96:3482–8. 10.3382/ps/pex117 28854744

[26] Saha A Perumalla A Lee Y Meullenet JF Owens CM. Tenderness, moistness, and flavor of pre- and postrigor marinated broiler breast fillets evaluated by consumer sensory panel. *Poult Sci.* (2009) 88:1250–6. 10.3382/ps.2008-00236 19439637

[27] Bowker BC Callahan JA Solomon MB. Effects of hydrodynamic pressure processing on the marination and meat quality of turkey breasts. *Poult Sci.* (2010) 89:1744–9. 10.3382/ps.2009-00484 20634532

[28] Peiretti P Gai F Rotolo L Brugiapaglia A Gasco L. Effects of tomato pomace supplementation on carcass characteristics and meat quality of fattening rabbits. *Meat Sci.* (2013) 95:345–51. 10.1016/j.meatsci.2013.04.011 23747628

[29] Kauffman R Eikelenboom G van der Wal PG Engel B Zaar M. A comparison of methods to estimate water-holding capacity in post-rigor porcine muscle. *Meat Sci.* (1986) 18:307–22. 10.1016/0309-1740(86)90020-3 22055735

[30] Xia X Kong B Liu J Diao X Liu Q. Influence of different thawing methods on physicochemical changes and protein oxidation of porcine longissimus muscle. *LWT Food Sci Technol.* (2012) 46:280–6.

[31] Chen L Zhou G Zhang W. Effects of high oxygen packaging on tenderness and water holding capacity of pork through protein oxidation. *Food Bioproc Tech.* (2015) 8:2287–97.

[32] Malila Y Petracci M Benjakul S Visessanguan W. Effect of tumbling marination on marinade uptake of chicken carcass and parts quality. *Rev Bras Cienc Avic.* (2017) 19:61–8.

[33] Kang D Gao X Ge Q Zhou G Zhang W. Effects of ultrasound on the beef structure and water distribution during curing through protein degradation and modification. *Ultrason Sonochem.* (2017) 38:317–25. 10.1016/j.ultsonch.2017.03.026 28633832

[34] Park D Xiong YL. Oxidative modification of amino acids in porcine myofibrillar protein isolates exposed to three oxidizing systems. *Food Chem.* (2007) 103:607–16.

[35] Fu Q Liu R Zhang W Li Y Wang J Zhou G. Effects of different packaging systems on beef tenderness through protein modifications. *Food Bioproc Tech.* (2015) 8:580–8.

[36] Culler R Smith G Cross H. Relationship of myofibril fragmentation index to certain chemical, physical and sensory characteristics of bovine longissimus muscle. *J Food Sci.* (1978) 43:1177–80.

[37] Thangavelu KP Kerry JP Tiwari BK Mcdonnell CK. Novel processing technologies and ingredient strategies for the reduction of phosphate additives in processed meat. *Trends Food Sci Technol.* (2019) 94:43–53. 10.1016/j.tifs.2019.10.001

[38] Baublits RT Pohlman FW Brown AH Johnson ZB. Effects of sodium chloride, phosphate type and concentration, and pump rate on beef biceps femoris quality and sensory characteristics. *Meat Sci.* (2005) 70:205–14. 10.1016/j.meatsci.2004.12.011 22063476

[39] Gao T Li JL Zhang L Jiang Y Liu Y Zhang X Effect of different tumbling marination methods and time on the quality characteristics of prepared pork chops. *Food Sci Technol.* (2015) 35:445–51.

[40] Yusop SM O’Sullivan MG Kerry JF Kerry JP. Influence of processing method and holding time on the physical and sensory qualities of cooked marinated chicken breast fillets. *LWT Food Sci Technol.* (2012) 46:363–70.

[41] Lopez K Schilling MW Armstrong TW Smith BS Corzo A. Sodium chloride concentration affects yield, quality, and sensory acceptability of vacuum-tumbled marinated broiler breast fillets. *Poult Sci.* (2012) 91:1186–94. 10.3382/ps.2011-01733 22499878

[42] Gao T Li JL Zhang L Jiang Y Ma R. Effect of different tumbling marination treatments on the quality characteristics of prepared pork chops. *Asian Australas J Anim Sci.* (2015) 28:260–7. 10.5713/ajas.14.0511 25557823 PMC4283172

[43] Anonymous. Tumbling and subsequent aging improves tenderness of beef steaks. *J Anim Sci.* (2022) 100:skac102. 10.1093/jas/skac102 PMC9017517 35438745

[44] Bertram HC Kohler A Backer U Ofstad R Andersen HJ. Heat-induced changes in myofibrillar protein structures and myowater of two pork qualities. a combined FT-IR spectroscopy and low-field NMR relaxometry study. *J Agric Food Chem.* (2006) 54:1740–6. 10.1021/jf0514726 16506827

[45] Kim HW Yan FF Hu JY Cheng HW Kim YHB. Effects of probiotics feeding on meat quality of chicken breast during postmortem storage. *Poult Sci.* (2016) 95:1457–64. 10.3382/ps/pew055 26944974

[46] Chandrapala J Zisu B Palmer M Kentish S Ashokkumar M. Effects of ultrasound on the thermal and structural characteristics of proteins in reconstituted whey protein concentrate. *Ultrason Sonochem.* (2011) 18:951–7. 10.1016/j.ultsonch.2010.12.016 21262585

[47] Riebroy S Benjakul S Visessanguan W Erikson U Rustad T. Acid-induced gelation of natural actomyosin from atlantic cod (*Gadus morhua*) and burbot (*Lota lota*). *Food Hydrocoll.* (2009) 23:26–39. 10.1016/j.foodchem.2007.12.008 26054263

[48] Estévez M. Protein carbonyls in meat system: a review. *Meat Sci.* (2011) 89:259–79. 21621336 10.1016/j.meatsci.2011.04.025

[49] Gülseren İ Güzey D Bruce BD Weiss J. Structural and functional changes in ultrasonicated bovine serum albumin solutions. *Ultrason Sonochem.* (2007) 14:173–83. 16959528 10.1016/j.ultsonch.2005.07.006

[50] Zhang RY Xing LJ Kang DC Zhou L Wang L Zhang WG. Effects of ultrasound-assisted vacuum tumbling on the oxidation and physicochemical properties of pork myofibrillar proteins. *Ultrason Sonochem.* (2021) 74:105582. 10.1016/j.ultsonch.2021.105582 33975184 PMC8122357

